# Bioactive Organocopper Compound from *Pseudomonas aeruginosa* Inhibits the Growth of *Xanthomonas citri* subsp. *citri*

**DOI:** 10.3389/fmicb.2016.00113

**Published:** 2016-02-09

**Authors:** Admilton G. de Oliveira, Flavia R. Spago, Ane S. Simionato, Miguel O. P. Navarro, Caroline S. da Silva, André R. Barazetti, Martha V. T. Cely, Cesar A. Tischer, Juca A. B. San Martin, Célia G. T. de Jesus Andrade, Cláudio R. Novello, João C. P. Mello, Galdino Andrade

**Affiliations:** ^1^Laboratório de Ecologia Microbiana, Departamento de Microbiologia, Universidade Estadual de LondrinaLondrina, Brazil; ^2^Laboratório de Espectroscopia, Departamento de Bioquímica e Biotecnologia, Universidade Estadual de LondrinaLondrina, Brazil; ^3^Laboratório de Microscopia Eletrônica e Microanálise, Departamento de Biologia Geral, Universidade Estadual de LondrinaLondrina, Brazil; ^4^Laboratório de Produtos Fitoterápicos, Departamento de Farmácia e Farmacologia, Universidade Estadual de MaringáMaringá, Brazil

**Keywords:** *Pseudomonas* secondary metabolites, citrus canker, purification process, antibiotic activity, bioactive compounds

## Abstract

Citrus canker is a very destructive disease of citrus species. The challenge is to find new compounds that show strong antibiotic activity and low toxicity to plants and the environment. The objectives of the present study were (1) to extract, purify and evaluate the secondary metabolites with antibiotic activity produced by *Pseudomonas aeruginosa* LV strain *in vitro* against *Xanthomonas citri* subsp. *citri* (strain 306), (2) to determine the potential of semi-purified secondary metabolites in foliar application to control citrus canker under greenhouse conditions, and (3) to identify antibiotic activity in orange leaf mesophyll infected with strain 306, by electron microscopy. Two pure bioactive compounds were isolated, an organocopper antibiotic compound (OAC) and phenazine-1-carboxamide. Phenazine-1-carboxamide did not show any antibiotic activity under the experimental conditions used in this study. The OAC showed a high level of antibiotic activity with a minimum inhibitory concentration of 0.12 μg mL^-1^. In greenhouse tests for control of citrus canker in orange trees, the semi-purified fraction F3d reduced lesion formation by about 97%. The concentration used was 500 times lower than that for the recommended commercial copper-based product. Electron microscopy showed that F3d altered the exopolysaccharide matrix and caused cell lysis of the pathogen inside the citrus canker lesions. These results suggest that secondary metabolites produced by inducing *P. aeruginosa* LV strain have a high potential to be used as a bioproduct to control citrus canker.

## Introduction

Brazil is one of the largest producers of orange juice concentrate in the world (Neves et al., 2010), earning over US$ 2 billion a year. However, citrus growers have suffered increasing losses mainly due to citrus canker and greening (Huanglongbing), where in the last decade, they were forced to destroy millions of trees (USDA, 2015). Citrus canker is one of the most destructive diseases of many commercial citrus species (Gottwald and Graham, 2000), and it is found in the biggest sweet orange producing areas, such as São Paulo State, Brazil, and Florida, USA. The etiological agent is *Xanthomonas citri* subsp. *citri* (synonyms *X. axonopodis* pv. *citri*, *X. campestris* pv. *citri*, and *X. citri* pv. *citri*), a Gram-negative, rod-shaped pathogenic bacterium belonging to the class Gammaproteobacteria (Gabriel et al., 1989; Schaad et al., 2005, 2006), and the genera of this family can infect over 350 species of plants (Chan and Goodwin, 1999).

Citrus canker causes premature fruit drop, defoliation, and shoot dieback. The infection begins by *X. citri* subsp. *citri* penetrating through natural openings (stomata) and wounds (Gottwald et al., 2002). Bacteria spread in orchards by wind-blown rain, and the colonization of leaf mesophyll increases with the presence of the citrus leaf miner *Phyllocnistis citrella* Stainton (Lepidoptera: Gracillariidae; Chagas et al., 2001). Growers use integrated management approaches such as cultivating less-susceptible citrus genotypes produced in *X. citri* subsp. *citri*-free nurseries, control of *P. citrella*, planting windbreaks as barriers to hinder the spread of the disease, and spraying synthetic copper bactericides (Schubert and Sun, 1996; Belasque and Behlau, 2011).

Today, research focuses on disease control in citrus crops in many ways, using systemic acquired resistance (SAR; Graham and Leite, 2004; Graham and Myers, 2009; Miller et al., 2011), obtaining more resistant citrus varieties (Fu et al., 2011; Yang et al., 2011), bacteriophages (Balogh et al., 2008) and *Pseudomonas* secondary metabolites (Góis et al., 2013; Spago et al., 2014; Murate et al., 2015). The low efficiency in controlling *X. citri* subsp. *citri* using commercially available compounds presents challenges in finding new techniques and/or products for control that are non-hazardous to the environment, have high bactericidal effects and are effective in infested internal tissues.

The potential of *Pseudomonas* species to suppress plant pathogens is well known (Dowling and O’Gara, 1994; Raaijmakers et al., 1997; Haas and Défago, 2005), and secondary metabolites produced by *Pseudomonas* show strong antibiotic activity, including phenazines, pyrrolnitrin-type antibiotics, pyo compounds, indole derivatives, peptides, glycolipids, lipids and aliphatic compounds (Fuller et al., 1971; Leisinger and Margraff, 1979; Ligon et al., 2000; Raaijmakers et al., 2002, 2006; Haas and Keel, 2003; Paulsen et al., 2005; Gross and Loper, 2009). *Pseudomonas* secondary metabolites can be an alternative source of new compounds for the control of plant diseases.

Given this background, the aims of our study were: (1) to extract, purify and evaluate the antimicrobial activity of secondary metabolites of *Pseudomonas aeruginosa* LV strain produced *in vitro* against *X. citri* subsp. *citri* (strain 306. Xcc 306), (2) to determine the potential of semi-purified secondary metabolites in foliar application to control citrus canker under greenhouse conditions, and (3) to identify the biological activity of semi-purified secondary metabolites inside the leaf, reducing the inoculum potential inside the citrus canker lesions by electron microscopy.

## Materials and Methods

### Chemicals and Media

All chemicals used for extraction and purification were of analytical grade. Silica gel and thin layer chromatography (TLC) plates were from Macherey–Nagel GmbH & Co. KG. Microbiological media were from Becton Dickinson and Company. Chemicals used for antimicrobial and cytotoxicity assays were purchased from Sigma–Aldrich. All other reagents were of analytical grade and the other chemicals used were of the highest purity.

### Bacterial Strains

The pathogen *X. citri* subsp. *citri* strain 306 (Xcc 306), whose genome has been sequenced (da Silva et al., 2002), was used in all experiments. Xcc 306 was stored in 40% glycerol (v/v) at –20°C, and the stock culture was renewed every 6 months and cultivated in nutrient agar (NA) at 28°C for 48 h. The antagonistic bacterium used was *P. aeruginosa* LV strain, which was isolated from an old citrus canker lesion on leaves of orange plants (*Citrus sinensis* cv. Valence), in the city of Astorga, Brazil (Rampazo, 2004). *P. aeruginosa* was maintained on NA plus copper chloride (CuCl_2_) (0.5% peptone, 0.3% meat extract, 0.01% CuCl_2_⋅2H_2_O and 1.5% agar; pH 6.8) at room temperature (28 ± 2°C) as the working culture. Glycerol stocks were also prepared and stored at –20°C throughout the study. The bacterial strains were deposited in the Microbial Culture Collection of the Laboratory of Microbial Ecology, Londrina State University, Brazil.

### Molecular Identification of Antagonistic Bacterium Strain LV

According to the result of a 16S rDNA partial base sequence and the API identification system, strain LV was closest to *P. aeruginosa*, where the 16S rDNA partial base sequence showed more than 99.9% similarity.

### Production and Purification of Metabolites with Antibiotic Activity

The culture parameters such as medium, pH, inoculum load, stirring, temperature, and culture age were standardized to optimize the growth of LV strains, and the production of metabolites was patented (Andrade, 2008). The initial inoculum of the LV strain was obtained from a culture stored in glycerol and cultivated as described above. When the culture was in log phase (10^8^ CFU mL^-1^, OD 0.09, λ = 590 nm) 100 μL of cell suspension was inoculated in 1 L of nutrient broth (NB) plus 100 mg L^-1^ CuCl_2_.2H_2_O and cultivated on a shaker for 10 days at 28°C and 100 rpm. The culture was harvested and centrifuged at 9,000 rpm for 20 min at 4°C. The metabolites were extracted five times from the cell-free culture supernatant using two volumes of dichloromethane each time (250 mL of supernatant and 500 mL of dichloromethane) and was designated the dichloromethane phase (DP). DP was purified by vacuum liquid chromatography (VLC). VLC was carried out in a glass column (35 cm long with diameter of 2 cm) filled with silica gel 60 (0.063–0.200 mm, Merck) coupled to a vacuum pump with ∼150 mmHg^-1^. DP was fractionated using the following mobile phase (v/v): hexane (F1), dichloromethane (F2), ethyl acetate (F3), methanol (F4), methanol/water (1:1; F5) and water (F6). Forty milliliters of each eluent was passed through the column 10 times, concentrated in a rotary evaporator (Rotavapor R 215, Büchi) under reduced pressure at 45°C. The fractions were monitored by TLC and antibiotic activity was identified by bioautography.

The F3 fraction (high level of antibiotic activity as shown in the bioautography test) was again purified by VLC as described above, except using the mobile phase (v/v): hexane (F3a), hexane/dichloromethane [1:1, (F3b)], dichloromethane (F3c), dichloromethane/ethyl acetate [1:1, (F3d)], ethyl acetate (F3e), ethyl acetate/methanol [1:1, (F3f)], methanol (F3g), methanol/water [1:1, (F3h)], and water (F3i). The fractions were monitored by TLC and antibiotic activity was identified by bioautography.

The F3d fraction (high level of antibiotic activity as shown in the bioautography test and yield of 150 mg per liter of culture) was again purified by flash chromatography. F3d was mixed with silica gel 60 (0.04–0.063 mm, Merck) to prepare the metabolite–silica gel slurry, which was air-dried until complete evaporation of the solvent at room temperature. The column (50 cm long with diameter of 0.8 cm) was coupled to a low-pressure pump and washed using a mobile phase (v/v) with different proportions of petroleum ether/dichloromethane/ethanol (68.5:30:1.5 and 50:40:10). Approximately 1 mL of the eluate was collected in tubes and monitored by TLC. Similar fractions were combined on the basis of TLC analysis and six combined fractions were obtained (F3d.1 to F3d.6). Antibiotic activity was identified by bioautography.

**FIGURE 1:**
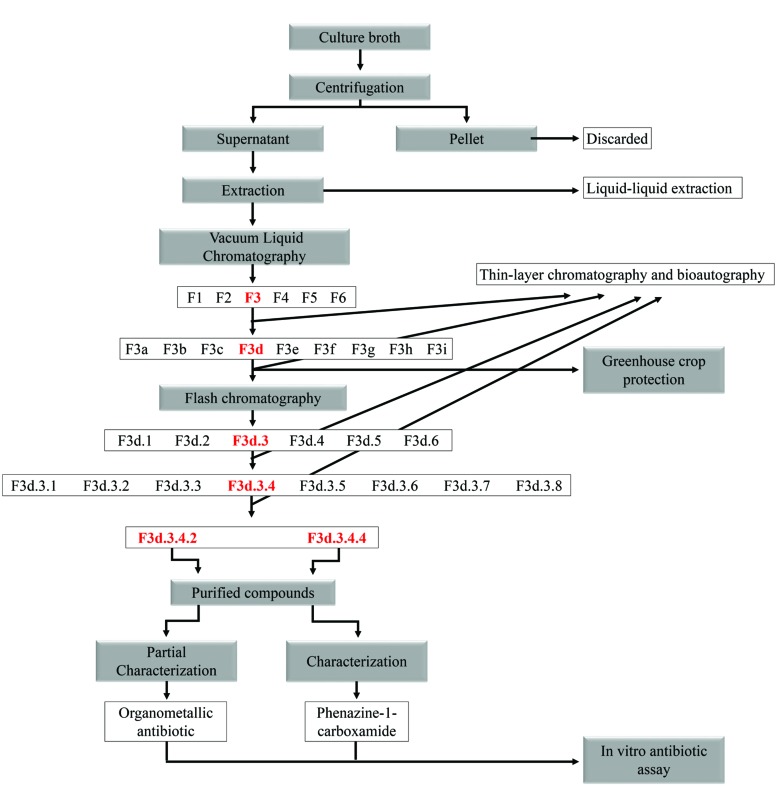
**Flow chart of purification of *Pseudomonas* secondary metabolites**.

The F3d.3 fraction was again purified by flash chromatography as described above, except the mobile phase was: petroleum ether/dichloromethane/ethanol (68.5:30:1.5), ethyl acetate and ethyl acetate/ethanol (1:1). Fractions of approximately 1 mL were collected in tubes and monitored by TLC. Similar fractions were combined on the basis of TLC analysis, and eight combined fractions were obtained (F3d.3.1 to F3d.3.8). Antibiotic activity was identified by bioautography. The F3d.3.4 fraction (high level of antibiotic activity as shown in the bioautography test) was again purified by flash chromatography as described above, except for the column size (20 cm long with diameter of 0.6 cm). Two pure compounds were obtained (F3d.3.4.2 and F3d.3.4.4), as summarized in a chart (**Figure 1**). One milligram of pure compounds was dissolved (chloroform and methanol, respectively) at room temperature and kept for slow evaporation in a vial. All pure compounds were then loaded on the high performance liquid chromatography (HPLC) column and were further subjected to chemical characterization.

### TLC Analysis

Thin layer chromatography of the fractions was carried out on silica gel plates (Merck 60 F_254_). The fractions were spotted on plates and after drying, the chromatograms were developed in dichloromethane/ethyl acetate/methanol (45:45:10) and/or ethyl acetate/methanol (50:50). The spots were viewed under ultraviolet light at 254 and 366 nm.

### HPLC Analysis

The pure compounds as obtained above were further subjected to HPLC using Agilent 1260 HPLC. The mobile phases used were 100% water (A) and 100% acetonitrile (B) at a flow rate 1 mL/min and injection volume was 100 μL at a column temperature of 25°C (C_18_ Agilent HPLC column, 5 μm, 4.6 mm × 250 mm). Eluate was monitored at different wavelengths (250, 264, 290, 271, 316, and 366 nm).

### Chemical Analysis

The pure compounds were dissolved in CDCl_3_ or CD_3_OD at 1,000 μg mL^-1^. Mass spectra were obtained with an ESI-MS Quattro LCZ (Micromass, Manchester, UK). ^1^H and ^13^C nuclear magnetic resonance spectra were recorded in solution using a Bruker Avance III 400 MHz spectrometer. X-ray microanalysis (EDS) was carried out using an FEI-Quanta 200 scanning electron microscopy with an accelerating voltage of 25 kV.

### Biological Analysis

#### Selection of Compounds with Antibiotic Activity by Bioautography

The bioautography method (Rahalison et al., 1993) was used to identify antibiotic activity. TLC plates were developed as described above and placed in a Petri dish with 20 mL of melted NA (45°C) mixed with 1 mL of cell suspension of Xcc 306 (10^8^ CFU mL^-1^), which was incubated for 48 h at 28°C. The zone of inhibition was used to identify the spots with compounds with antibiotic activity.

#### Disk Diffusion Method

The disks were prepared with different concentrations of semi-purified fractions and purified compounds with antibiotic activity (1,000 μg disk^-1^ with the DP extract and F3 fraction; 100 μg disk^-1^ of F3d and F3d.3 fractions; 30 μg disk^-1^ of F3d.3.4 fraction and F3d.3.4.2, F3d.3.4.4 purified compounds), with three replicates. The disks were placed on a Petri dish with 20 mL of NA previously inoculated with a cell suspension of Xcc 306 (10^8^ CFU mL^-1^) in the log phase. The solvent, dichloromethane, was considered the negative control. Plates were incubated for 48 h at 28°C. The experiment was repeated three times and the antibiotic effect was determined by measuring (mm) the inhibition halos formed around the disks.

#### Minimum Inhibitory Concentration (MIC)

Xcc 306 was grown overnight in NB at 28°C, and MIC was determined in 96-well plates using twofold serial dilutions. The concentrations tested ranged 2,500 to 4.88 μg mL^-1^ for DP extract and F3 fraction, 400 to 0.78 μg mL^-1^ for F3d fraction and 2 to 0.004 μg mL^-1^ for F3d.3.4.2 purified compound. The inoculum used was 100 μL of cell suspension of Xcc 306 (10^7^ CFU mL^-1^) per well. The growth control was a cell suspension of Xcc 306 in NB only, and the negative control was non-inoculated NB. The antibiotic solution was mixed with NB to check for sterility. Plates were incubated for 48 h at 28°C. The optical density was determined at 590 nm (BioMate 3). Afterward, cell viability was determined, where 20 μL of 1% 2, 3, 5-triphenyltetrazolium chloride were added to all wells and the plates incubated again at 28°C for 20 min. Afterward, the wells that showed a pink color indicated resistance (+), while no color change indicated sensitivity (–). The experiment was repeated three times.

#### Cytotoxicity Assay

The LLCMK2 cell line was grown in 96-well microplates (Techno Plastic Products, Switzerland) containing RPMI medium supplemented with 10% bovine serum, at a density of 2.5 × 10^4^ cells/well for 24 h in 5% CO_2_, 37°C. At confluence, non-adherent cells were removed by washing with sterile 0.15 M phosphate-buffered saline pH 7.2 (PBS). Afterward, twofold serial dilutions of F3d with antibiotic activity were made (2000 to 1 μg mL^-1^) and added to each well containing the cells. The plates were incubated for 72 h at 37°C in 5% CO_2_. Cell viability was determined by the dimethylthiazol-diphenyl-tetrazolium-bromide [MTT – (Sigma Chemical Co, USA)] method according to the manufacturer’s recommendations. Wells containing medium alone or medium plus 1% DMSO served as growth and sterility controls. The concentration of F3d needed to reduce cell viability up to 50% by regression analysis corresponded to the 50% cytotoxic concentration (CC_50_).

#### Foliar Application to Control Citrus Canker Under Greenhouse Conditions

Plants of *C. sinensis* cv. Valence were grown in 3-L pots with non-sterile rhodic ferralsol soil (FAO, 1994) in a greenhouse (28°C/22°C and 10 h/14 h day/night period, 80% relative humidity). Every 15 days, 150 mL of Hewitt’s solution for non-legume plants (Hewitt, 1966) were added, and plants were watered with tap water when needed. The experiments were carried out in a completely randomized block design using two regimens, preventive and curative, and three doses of F3d according to the MIC (D1 = 1 μg mL^-1^; D2 = 10 μg mL^-1^; and D3 = 100 μg mL^-1^) with five replicates of each treatment (2 × 3 × 5). Positive control plants were sprayed with Xcc 306 cell suspension and negative control plants with distilled water. Xcc 306 infection was carried out with plants kept in a humid chamber, where plants were covered with 150 L black plastic bags for 24 h at 30°C to maintain high humidity and stoma opening, before and after spraying bacterial suspension or antibiotic solution. Two spray times were used to test for curative or preventive effect against citrus canker. In the preventive regimen, 8 mL of F3d fraction were sprayed per plant 24 h before spraying with 8 mL of Xcc 306 (10^8^ CFU mL^-1^). In the curative regimen, plants were sprayed first with Xcc 306 and F3d 24 h later. Negative controls were sprayed with 8 mL of distilled water (the solvent used to dilute F3d). The number of lesions was determined after 21 days of last spraying. Considering the high lesion formation and homogeneity in control plants, the number of lesions in an area was determined with a stereomicroscope (40×) and accordingly multiplied to obtain the number for the whole leaf area. Data were analyzed with SigmaPlot software and quadratic regression, where *p* ≤ 0.01 was considered significant.

#### Ultrastructural Evaluation

Samples of orange leaf (6 mm in diameter) were collected during the plant experiment under greenhouse conditions (24 and 120 h) in both treatment regimens (curative and preventive) at a dose of 10 μg mL^-1^. For SEM analysis, samples collected at 24 h were fixed by immersion in 2.5% glutaraldehyde, 2% paraformaldehyde in 0.1 M sodium cacodylate buffer, post-fixed in 1% OsO_4_, and dehydrated in an ethanol series (70, 80, 90, and 100%). Samples were critical-point dried with CO_2_ (BALTEC CPD 030 Critical Point Dryer), coated with gold (BALTEC SDC 050 Sputter Coater) and observed under a scanning electron microscope (FEI Quanta 200). For transmission electron microscopy (TEM) analysis, samples collected at 120 h were fixed and dehydrated as described above and were embedded and blocked in Araldite^®^ resin. Ultrathin sections (60–70 nm) were stained with uranyl acetate and lead citrate and observed under a TEM (FEI Tecnai 12).

## Results

### Purification, Crystallization, and Characterization of Purified Compounds

Two major metabolites were isolated from F3d fraction, and crystals were analyzed after complete evaporation. The first pure compound (F3d.3.4.2) showed a single peak at UV absorption of 271 nm and high antibiotic activity (**Supplementary Figure S1**). F3d.3.4.2 appeared as low-quality dark-green crystals after evaporation. It was completely soluble in DMSO and CDCl_3_. The molecular structure has not yet been completely determined. On the basis of the spectral data, the bioactive compound was determined to be a natural organocopper antibiotic compound (OAC; **Supplementary Figures S2**–**S4**). The second one (F3d.3.4.4) appeared as high-quality fine yellow crystals. It was completely soluble in DMSO and CD_3_OD. ESI-MS and NMR analysis of this bioactive compound was close to that proposed by Naik and Sakthivel (2006), phenazine-1-carboxamide (PCN), with the molecular formula C_13_H_9_N_3_O (**Supplementary Figures S5**–**S8**).

### Evaluation of *In vitro* Antibiotic Activity

Whole purification steps of the DP extract were monitored by bioautography until pure compounds were obtained, and only the fractions F3, F3d, F3d.3, F3d.3.4, and OAC (F3d.3.4.2) showed significant antimicrobial activity. The other fractions and PCN did not show any antibiotic activity under the experimental conditions used in this study.

**FIGURE 2:**
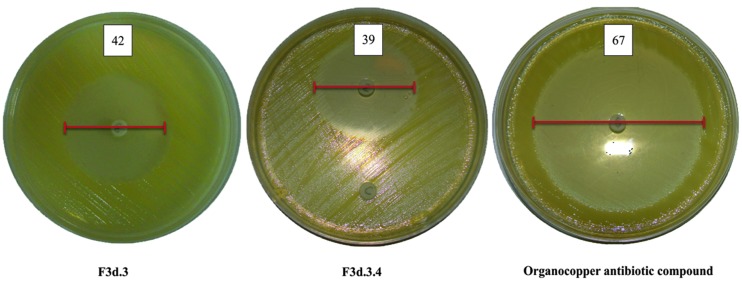
**Effect of *Pseudomonas* secondary metabolites on the growth of Xcc 306**.

As the purification process proceeded, the antibiotic activity against Xcc 306 increased, where DP extract showed a lower inhibitory effect in the disc diffusion assay (21.5 mm at 1,000 μg disk^-1^) when compared with more purified fractions: F3 (26.5 mm at 1,000 μg), F3d (32 mm at 100 μg), F3d.3 (42 mm at 100 μg), and F3d.3.4 (39 mm at 30 μg). The activity increased further when testing the OAC, which showed an inhibition halo of 67 mm at 30 μg mL^-1^ against Xcc 306 (**Table 1** and **Figure 2**).

**Table 1:** Antibiotic activity against Xcc 306 by disk diffusion technique of fractions and pure compound obtained from purification of extract containing secondary metabolites produced by *Pseudomonas aeruginosa* LV strain.

Concentration (μg disk^-1^)	Fractions					Purified compounds	
		
	FD	F3	F3d	F3d.3	F3d.3.4	F3d.3.4.2 (OAC)	F3d.3.4.4 (PCN)
**Inhibition zone diameter (mm)**							
1,000	21.5 ± 1	26.5 ± 1	^∗^	^∗^	^∗^	^∗^	^∗^
100	^∗^	^∗^	32 ± 0.5	42 ± 1	^∗^	^∗^	^∗^
30	^∗^	^∗^	^∗^	^∗^	39 ± 0.5	67 ± 0.5	-
NC	-	-	-	-	-	-	-


*Test substances were previously selected by bioautography. Solvent was used as the negative control. The data represent the mean ± standard deviation of three replicates. Negative control (NC); no tested (^∗^); absence of zone of inhibition (–); organocopper antibiotic compound (OAC); phenazine-1-carboxamide (PCN).*

The same results were observed in the MIC assay against Xcc 306, where DP, F3, and F3d were 78.12, 19.53, and 6.25 μg mL^-1^, respectively. OAC showed a high level of antibiotic activity with a MIC of 0.12 μg mL^-1^ (**Table 2**).

**Table 2:** Determination of minimum inhibitory concentration of fractions and pure compounds obtained from secondary metabolites produced by *Pseudomonas aeruginosa* LV strain, against Xcc 306.

Sample	MIC (μg mL^-1^)
DP	78.12
F3	19.53
F3d	6.25
F3d.3.4.2 (OAC)	0.12
NC	–


*Negative control (NC); dichloromethane phase (DP); no antibiotic activity (–); organocopper antibiotic compound (OAC).*

F3d was chosen to use in the greenhouse experiments for many reasons, namely good availability, strong antibiotic activity and because it was mostly composed of OAC (30%), where it was known that OAC was the only component with high antibiotic activity.

### Cytotoxicity Assay

It was not possible to determine the 50% cytotoxic concentration of F3d to LLC-MK2 cells, since with the highest concentration tested (2000 μg mL^-1^), around 84% against the mammalian cells were viable, according to the MTT assay.

### Foliar Application to Control Citrus Canker Under Greenhouse Conditions

**FIGURE 3:**
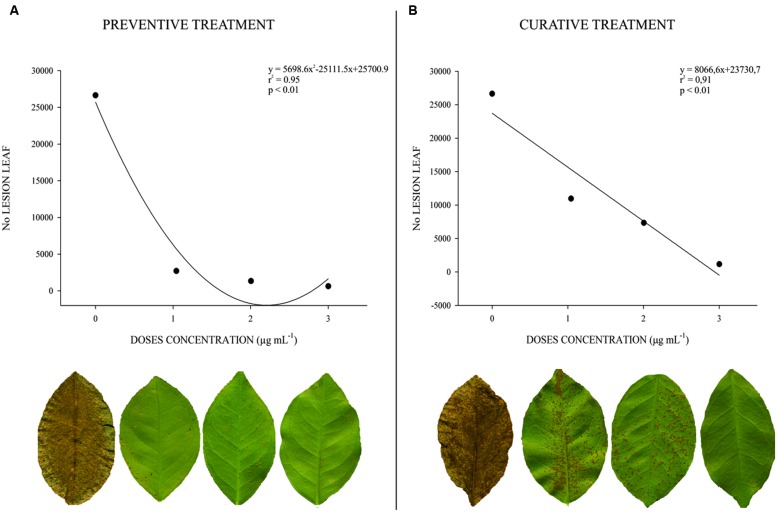
**Determination of dose–effect relation for F3d semi-purified fraction against the number of lesions of citrus canker caused by Xcc 306 strain in leaf of *C. sinensis* cv. Valence, 21 days after application (*n* = 50).** (0 = Xcc 306 suspension not treated; 1 = 1 μg mL^-1^; 2 = 10 μg mL^-1^; 3 = 100 μg mL^-1^. **(A)** Preventive treatment. **(B)** Curative treatment.

The preventive treatment showed a high correlation between dose and lesion number (*r*^2^ = 0.95; *p* < 0.01). All doses of F3d decreased lesion number when compared with untreated plants: 1 μg mL^-1^ = 90%, 10 μg mL^-1^ = 93%, and 100 μg mL^-1^ = 97% (**Figure 3A**). The curative treatment showed the same correlation (*r*^2^ = 0.97; *p* < 0.01), but lesion number was higher when compared with the preventive treatment, two times at 1 μg mL^-1^ and three and half times at 10 and 100 μg mL^-1^ (**Figure 3B**).

### Ultrastructural Evaluation

**FIGURE 4:**
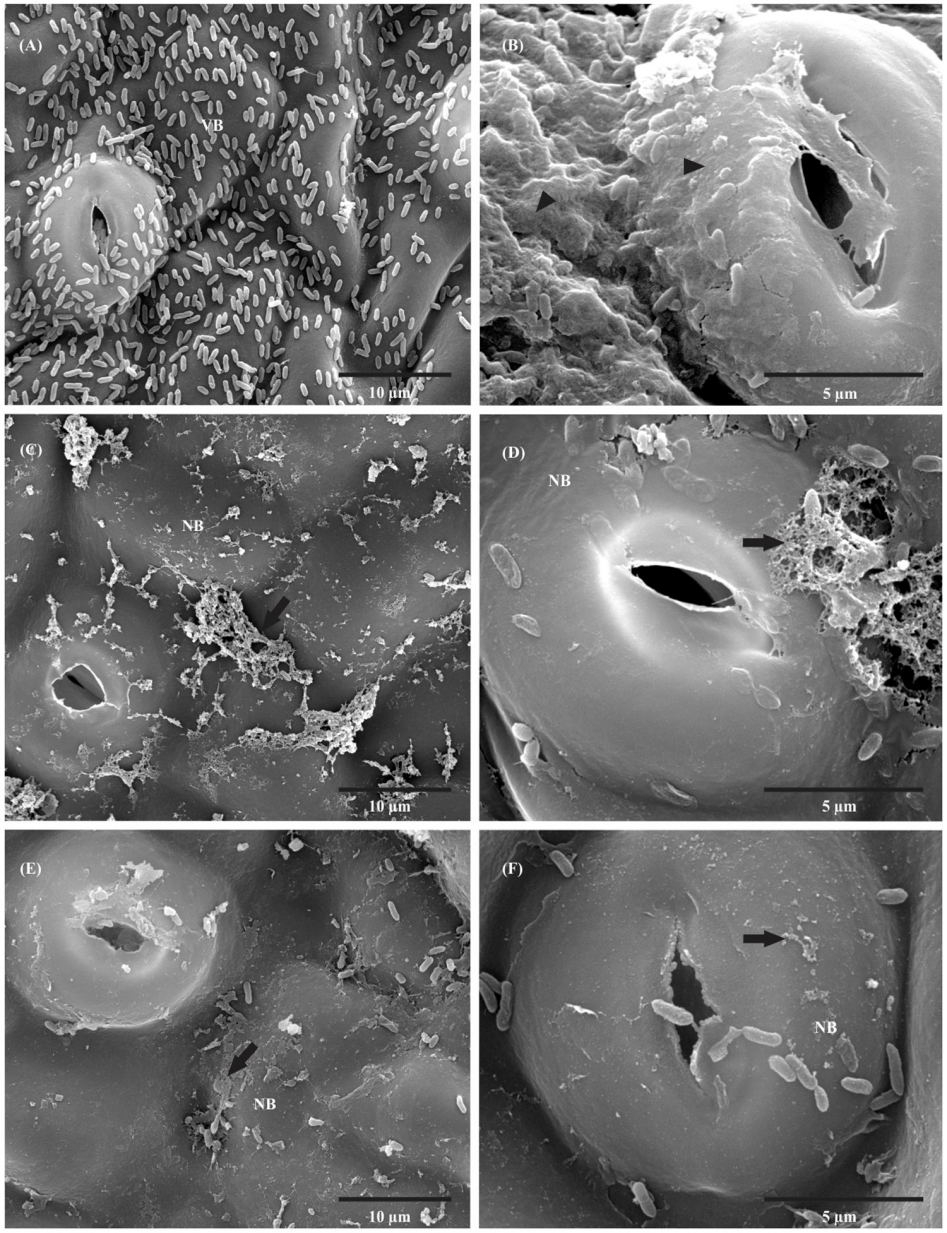
**Scanning electron micrographs of orange leaf (*C. sinensis* cv. Valence) inoculated with 10^8^ CFU mL^-1^ Xcc 306.** **(A)** Control (not treated with F3d) 24 h after inoculation; large number of Xcc 306 present on leaf surface with intact appearance. **(B)** Higher magnification of control; there is a thick layer of extracellular polysaccharides (EPS) on the leaf surface. **(C)** Preventive treatment 24 h after F3d application, showing a decreased number of bacteria and presence of non-viable Xcc 306 and altered EPS. **(D)** Higher magnification of preventive treatment; observed morphological changes in bacterial shape and EPS. **(E)** Curative treatment 24 h after F3d application, showing a decreased number of bacteria and morphological changes in bacterial shape and EPS. **(F)** Higher magnification of curative treatment; observed morphological changes in bacterial shape and EPS absence. (VB, viable cell; NB, non-viable cells; Arrowhead = Xcc 306 embedded in amorphous EPS; Arrow = changed in the configuration of EPS).

In SEM, a large number of Xcc 306 cells were observed on the leaf surface and they appeared intact and embedded in amorphous extracellular polysaccharides (EPS) 24 h after inoculation (**Figures 4A,B**). With preventive (**Figures 4C,D**) and curative (**Figures 4E,F**) treatments, the number of bacteria decreased, and morphological changes in bacterial shape and EPS were observed after 24 h.

**FIGURE 5:**
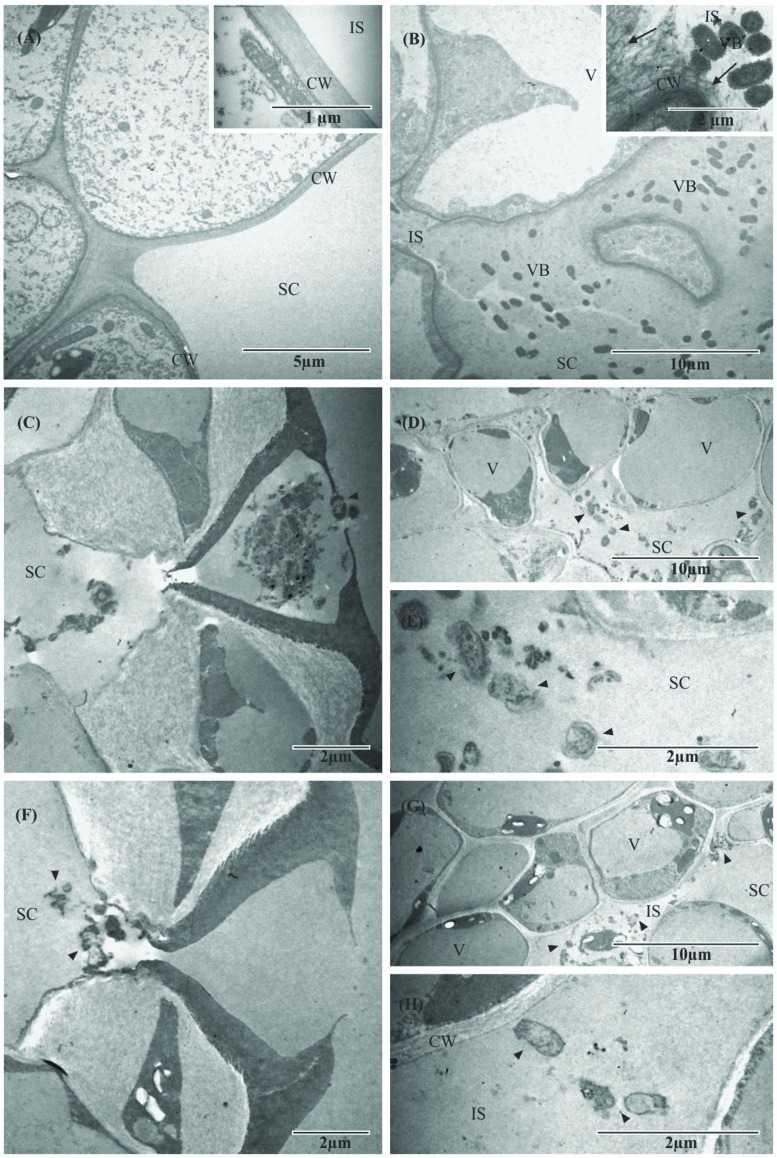
**Transmission electron micrographs of orange leaf (*C. sinensis* cv. Valence) inoculated with 10^8^ CFU mL^-1^ Xcc 306.** **(A)** Leaf of non-inoculated plant, showing complete absence of Xcc 306 cells and EPS in the intercellular space of mesophyll cells. Inset shows an intact cell wall at higher magnification. **(B)** Control not treated with F3d 120 h after inoculation, showing the intercellular space colonized with Xcc 306 and EPS evidenced by the electron-dense appearance. Inset shows viable bacterial cells present in the intercellular space in contact with host cell wall and disruption process at higher magnification. **(C)** The 120 h after inoculation in the preventive treatment; non-viable Xcc 306 cells are shown at the entrance of outer stomatal chamber and substomatal chamber. **(D)** After 120 h of preventive treatment, few bacterial cells are seen in the leaf mesophyll, with altered bacterial cell morphology and different electron-dense appearance in intercellular space (suggesting absence of EPS). **(E)** After 120 h of inoculation in the preventive treatment, higher magnification of **Figure 4D**, showing the presence of non-viable Xcc 306 cells in the substomatal chamber. **(F)** After 120 h in the curative treatment, the same effects are seen when compared with preventive treatment on orange leaves, including the effect on bacterial cells. **(G)** Mesophyll cells are unaltered and bacterial cells (Xcc 306) appear lysed, with different electron-dense appearance in intercellular space (suggesting absence of EPS). **(H)** Presence of non-viable Xcc 306 cells in the intact mesophyll cell and absence of EPS at higher magnification of **Figure 4F**. (CW, host cell wall; IS, intercellular space; SC, substomatal chamber; VB, viable cell; V, vesicle; arrowhead, non-viable Xcc 306 cell).

In TEM, the negative control (non-inoculated leaf) showed no change in the mesophyll, which appeared to have intact cell walls, with the substomatal chamber having an electron-lucid appearance, after 120 h (**Figure 5A**, inset). In the positive control (infected and untreated plant), viable bacteria were frequently observed in the intercellular spaces and substomatal chamber of the leaf, and plant cell organelles appeared damaged. The intracellular space and the substomatal chamber were full of EPS evidenced by its electron-dense appearance (**Figure 5B**, inset).

After 120 h in the preventive treatment, there were few bacterial cells in the leaf mesophyll, altered bacterial cell morphology, and differences in electron-dense aspect of the substomatal chamber (suggesting absence of EPS), and the mesophyll cells were unaltered, while the bacterial cells (Xcc 306) appeared lysed (**Figures 5C–E**). After 120 h in the curative treatment, the same effects were found in orange leaves when compared with the preventive treatment, including the effect on bacterial cells (**Figures 5F–H**).

## Discussion

Previous studies have shown the antibiotic effect of *Pseudomonas* secondary metabolites against various bacterial species (de Oliveira et al., 2011; Lopes et al., 2012; Cardozo et al., 2013; Spago et al., 2014). In the present study, we evaluated the effect of F3d, a semi-purified fraction, on plants infected by Xcc 306, evaluating antibiotic effectiveness in the initial infection process on the leaf and the antibiotic control of Xcc 306 infecting the mesophyll tissue inside the lesions, and we determined the active pure compound present in the F3d fraction (30% OAC). The product used in this study, showed about 90% reduction in citrus canker lesion formation in orange plants at a concentration of 1 μg mL^-1^, a concentration about 200 times lower than that for the recommended commercial copper-based compound, and more active than other products tested for control of citrus canker (Silva et al., 2013).

The observed antibiotic effect of F3d on citrus canker differed when comparing preventive and curative regimens, where the preventive was more effective in the control of lesion formation and number of bacteria on leaf surface in the mesophyll inside the lesion.

The differences observed between preventive and curative regimens suggested that when the plants were inoculated with Xcc 306 after F3d application (preventive), F3d reduced the bacterial population during the first hours, which was also observed in *in vitro* experiments. In this way, the substantial control of Xcc 306 observed in a preventive treatment was likely related to mechanisms other than antibiotic activity such as SAR. The preventive effect was also observed in other studies with different species of *Xanthomonas* and host plants (Lopes et al., 2012; Spago et al., 2014; Vasconcellos et al., 2014). Phenazine can influence growth and induce SAR in plants (Pierson and Pierson, 2010), and is produced by many bacterial species. Different phenazines are produced by the same bacterial species but in different proportions and with different activities (Pierson and Pierson, 2010). The PCN isolated in this study did not show antibiotic activity. On the other hand, PCN bioactivity in citrus canker control could be related to the induction of resistance mechanisms, which needs to be elucidated in further studies.

By electron microscopy, it was possible to observe the bacteriolytic activity of F3d in treated plants compared with untreated plants. The infection of orange leaves by Xcc 306 in untreated plants caused changes in the mesophyll cells, where fibrillar material was found in the cell walls leaking into the intercellular space. Also, viable bacterial cells were frequently observed in the intercellular spaces, and the substomatal chamber was filled with homogeneous EPS. On the other hand, treated plants showed non-viable bacterial cells in the mesophyll (lysed or disintegrated bacterial cells), and mesophyll morphology and cell walls remained unaltered, suggesting that F3d acted inside the leaf and did not have a phytotoxic effect at all concentrations tested.

Transmission electron microscopy showed the absence of EPS in the intercellular space in infected *C. sinensis* Valence leaves treated with F3d. This result corroborated the findings of de Oliveira et al. (2011), who demonstrated that *Pseudomonas* crude secondary metabolites not only possessed antibiotic activity but also altered bacterial cell morphology and reduced the presence of EPS in an *in vitro* study. Considering these results and also the finding of 97% reduction in the number of lesions in treated plants, F3d had two activities: it killed the bacteria and affected the formation of EPS.

The function of EPS in *Xanthomonas* species is controversial, and many authors have reported that EPS production is associated with the virulence level of many *Xanthomonas* species (Katzen et al., 1998; Dharmapuri and Sonti, 1999; Kemp et al., 2004). On the other hand, authors consider that EPS does not play an essential role in citrus canker in the initial stages of infection but facilitates the maintenance of bacteria in the host plant, possibly improving the efficiency of colonization in deep tissue of the leaf (Dunger et al., 2007). According to Rigano et al. (2007), in *X. axonopodis* pv. *citri*, xanthan is essential for the formation of microcolonies and the subsequent development of more complex structures, in which bacteria are tightly packed in hexagonal arrays separated by water-filled channels. Such complex structures are found both *in vitro* and *in planta*. In addition, the same authors reported in their studies that the presence of plant-associated biofilms was correlated with *X. axonopodis* pv. *citri* pathogenicity, which was markedly attenuated by the disruption of a single gene involved in the biosynthesis of EPS.

The present study showed that semi-purified secondary metabolites (F3d) had strong antibiotic activity without phytotoxicity to orange plants, and that activity persisted for many weeks on the phylloplane and inside the leaf, reducing the inoculum potential outside and inside the citrus canker lesions. F3d has great potential in the near future for use in the field to control citrus canker disease, but more field studies are needed first.

## Author Contributions

All authors listed, have made substantial, direct and intellectual contribution to the work, and approved it for publication.

## Conflict of Interest Statement

The authors declare that the research was conducted in the absence of any commercial or financial relationships that could be construed as a potential conflict of interest.
