# Supplementary material for: Bioactive Organocopper Compound from Pseudomonas aeruginosa Inhibits the Growth of Xanthomonas citri subsp. citri
Source: Front Microbiol. 2016 Feb 9;7:113. doi: 10.3389/fmicb.2016.00113 (PMC4746251; doi:10.3389/fmicb.2016.00113)
Supplement: Supplementary file 1 [file Data_Sheet_1.DOCX]

***Supplementary Material***

**Bioactive organocopper compound from *Pseudomonas aeruginosa* inhibits the growth of *Xanthomonas citri* subsp. *citri***

**Admilton Gonçalves de Oliveira^1^, Flavia Regina Spago^1^, Ane Stefano Simionato^1^, Miguel Octavio Pérez Navarro^1^, Caroline Santos da Silva^1^, André Riedi Barazetti^1^, Martha Viviana Torres Cely^1^, Cesar Augusto Tischer^2^, Juca Abramo Barrera San Martin^3^, Célia Guadalupe Tardeli de Jesus Andrade^3^, Cláudio Roberto Novello^4^, João Carlos Palazzo de Mello^4^, Galdino Andrade^1*^**

^1^Laboratório de Ecologia Microbiana, Departamento de Microbiologia, Universidade Estadual de Londrina, Londrina, Paraná, Brazil.

^2^Laboratório de Espectroscopia, Departamento de Bioquímica e Biotecnologia, Universidade Estadual de Londrina, Londrina, Paraná, Brazil.

^3^Laboratório de Microscopia e Microanálise, Departamento de Biologia Geral, Universidade Estadual de Londrina, Londrina, Paraná, Brazil.

^4^Laboratório de Produtos Fitoterápicos, Departamento de Farmácia e Farmacologia, Universidade Estadual de Maringá, Maringá, Paraná, Brazil.

*Corresponding author: Galdino Andrade, Universidade Estadual de Londrina, Centro de Ciências Biológicas, Departamento de Microbiologia. CEP 86051-990, Londrina, Brazil.

andradeg@uel.br

**Supplementary Figures**


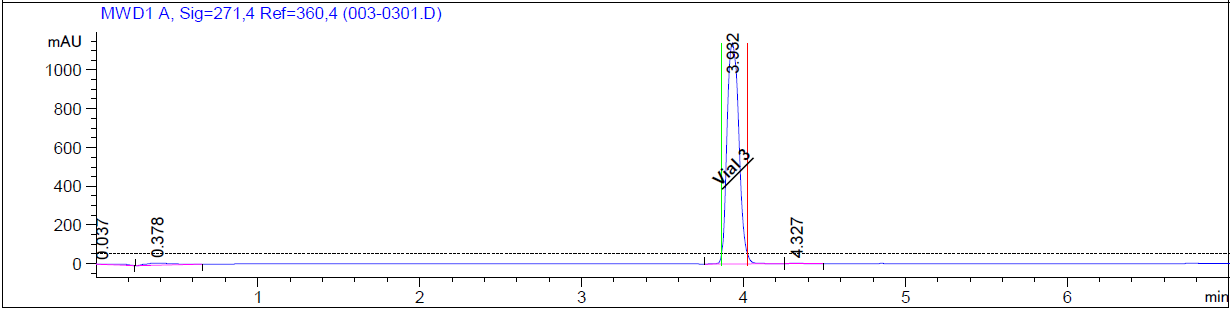


**Fig. S1** HPLC chromatogram of the natural organocopper antibiotic compound (F3d.3.4.2) produced by *Pseudomonas aeruginosa* LV strain.


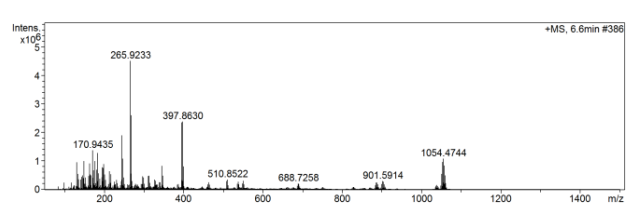


**Fig. S2** ESI-MS (positive mode) of the natural organocopper antibiotic compound (F3d.3.4.2) produced by *Pseudomonas aeruginosa* LV strain.


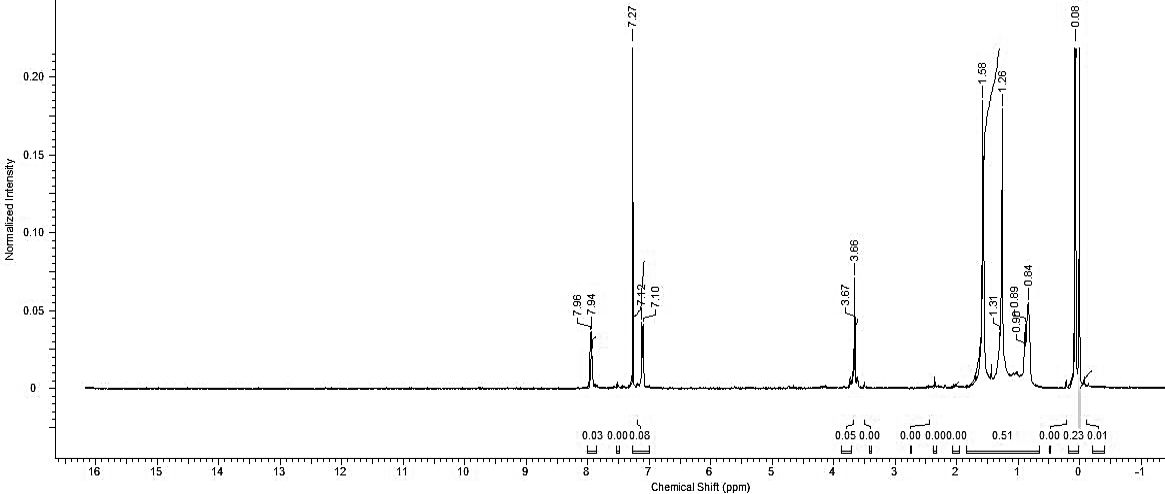


**Fig. S3** ^1^H NMR of the natural organocopper antibiotic compound (F3d.3.4.2) produced by *Pseudomonas aeruginosa* LV strain (300 MHz; CDCl_3_).


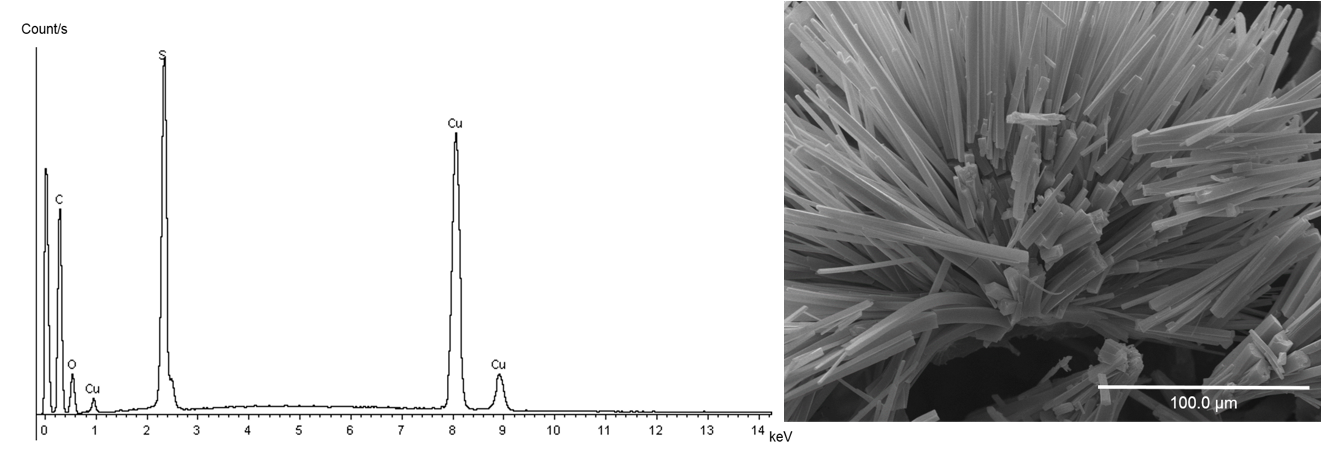
**Fig. S4** SEM - EDS spectrum of the natural organocopper antibiotic compound (F3d.3.4.2) produced by *Pseudomonas aeruginosa* LV strain.

**Fig. S5** ESI-MS of the phenazine-1-carboxamide (F3d.3.4.4) produced by *Pseudomonas aeruginosa* LV strain.


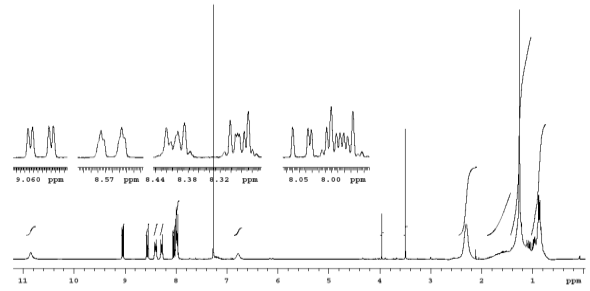


**Fig. S6** ^1^H NMR of the phenazine-1-carboxamide (F3d.3.4.4) produced by *Pseudomonas aeruginosa* LV strain (300 MHz; CD_3_OD).


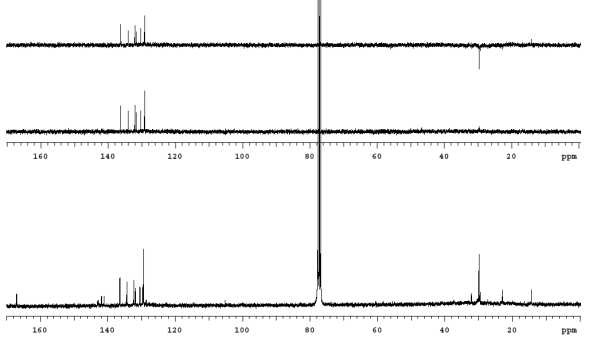
**Fig. S7** ^13^C NMR of the phenazine-1-carboxamide (F3d.3.4.4) produced by *Pseudomonas aeruginosa* LV strain (75,5 MHz; CD_3_OD).


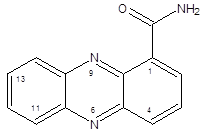


**Fig. S8** Molecular structures of the phenazine-1-carboxamide (F3d.3.4.4) produced by *Pseudomonas aeruginosa* LV strain
